# Sudden circulatory collapse caused by mechanical obstruction of the left main coronary trunk with infective endocarditis vegetation: a case report

**DOI:** 10.1186/s40792-021-01296-3

**Published:** 2021-10-14

**Authors:** Rena Usui, Masato Mutsuga, Yuji Narita, Yoshiyuki Tokuda, Sachie Terazawa, Hideki Ito, Wataru Uchida, Akihiko Usui

**Affiliations:** Department of Cardiac Surgery, Nagoya University Graduate School of Medicine, 65 Tsurumai-cho, Showa-ku, Nagoya, Aichi Japan

**Keywords:** Coronary artery obstruction, Infective endocarditis, Acute coronary syndrome

## Abstract

**Background:**

Acute coronary syndrome (ACS) caused by mechanical obstruction of the coronary artery with a vegetation is extremely rare but associated with high mortality. The optimal management strategy of this condition remains controversial because of its scarcity. We experienced a case of sudden circulatory collapse due to mechanical occlusion of the left main coronary trunk with a vegetation.

**Case presentation:**

A 68-year-old woman with aortic and mitral valve infective endocarditis suffered sudden dyspnea followed by heart arrest while awaiting surgery. Despite treatment with adequate antibiotic therapy, she had had multiple embolic infarctions and ruptured infectious cerebral aneurysms. We conducted transcatheter arterial embolization of the aneurysm and postponed the cardiac surgery due to residual aneurysmal blood flow. She suffered sudden cardiac arrest, and extracorporeal membrane oxygenation was applied after cardiopulmonary resuscitation. An echocardiogram revealed diffuse severe hypokinesis, and emergency coronary angiography was performed under suspicion of ACS. It revealed obstruction of the left main coronary trunk by a vegetation. Emergent cardiac surgery was performed. A vegetation had occluded the left coronary orifice. Aortic and mitral valve replacement with coronary artery bypass to the left antero-descending branch was performed. Regarding her cardiac function, she still required extracorporeal membrane oxygenation after surgery. She passed away 19 days after surgery due to multiple organ failure.

**Conclusions:**

ACS caused by mechanical obstruction of the coronary artery with a vegetation is rare but associated with high mortality. When circulatory collapse acutely occurs in patients with aortic valve infective endocarditis, we should suspect acute coronary artery obstruction. Urgent coronary angiography is mandatory to rescue the patient while preparing for emergency surgery.

## Background

Acute coronary syndrome (ACS) is a rare complication of infective endocarditis (IE). ACS caused by mechanical obstruction of the coronary artery with a vegetation is extremely rare but associated with high mortality and morbidity rates. The optimal management strategy of this condition remains controversial because of its scarcity.

We herein report a case of IE with sudden circulatory collapse due to mechanical occlusion of the left main coronary trunk with a vegetation.

## Case presentation

A 68-year-old woman had had a fever for 18 days after total hysterectomy for repeat urinary tract infection *by Enterococcus faecalis* due to uterine prolapse. An echocardiogram revealed a large mobile vegetation at both the aortic and mitral valves with severe regurgitation. Her blood cultures were positive for *Enterococcus faecalis*. Cerebral magnetic resonance imaging (MRI) revealed asymptomatic small embolic infarction at the right middle cerebral artery region. She was treated with adequate antibiotic therapy, and her heart failure was abated with medication.

She was transferred to our hospital for surgery for endocarditis 18 days after the administration of antibiotics.

Follow-up cerebral MRI revealed multiple new embolic infarctions and ruptured infectious aneurysms at the left posterior cerebral artery. We conducted transcatheter arterial embolization of the aneurysm and postponed the cardiac surgery due to residual aneurysmal blood flow. *Follow-up echocardiogram revealed no change of vegetation size and severeness of regurgitation.*

**Fig. 1:**
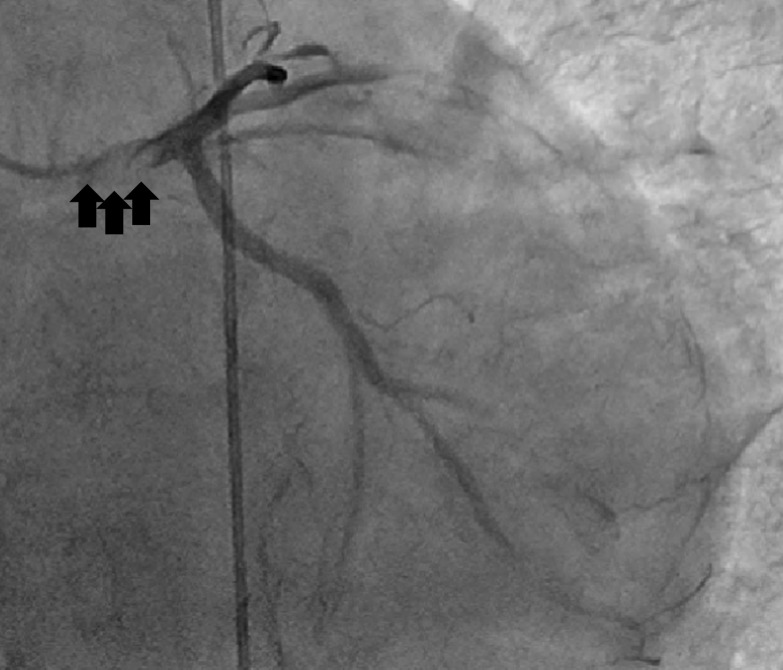
Coronary angiography revealed obstruction of the left main coronary trunk by a vegetation (black arrow)

While awaiting surgery for 3 weeks, she suffered sudden dyspnea followed by cardiac arrest. Extracorporeal membrane oxygenation was applied after cardiopulmonary resuscitation. An echocardiogram revealed diffuse severe hypokinesis, and emergency coronary angiography was performed under suspicion of ACS. It revealed obstruction of the left main coronary trunk by a vegetation (Fig. 1).

**Fig. 2:**
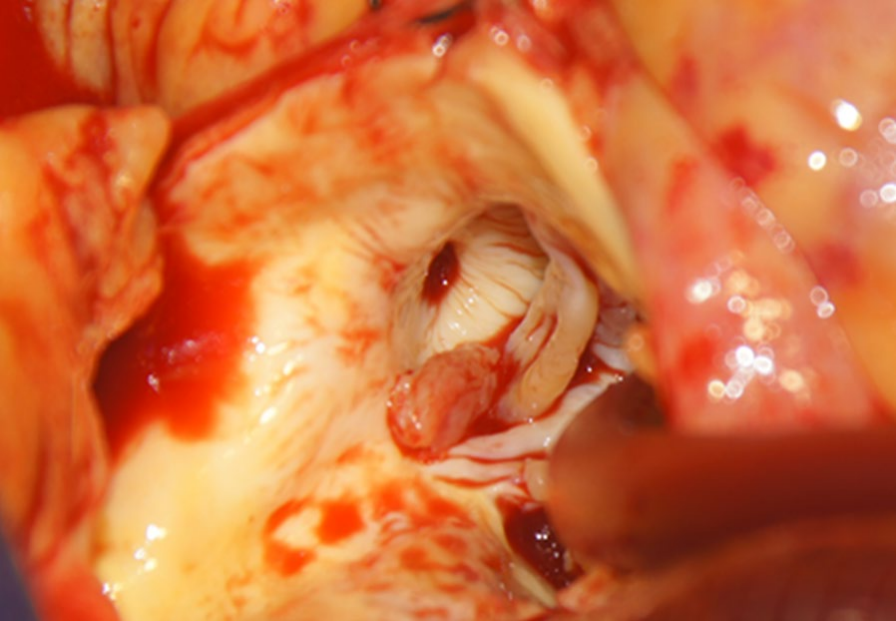
Intraoperative findings: a 3 × 6-mm aortic vegetation was found to have occluded the left main coronary trunk

**Fig. 3:**
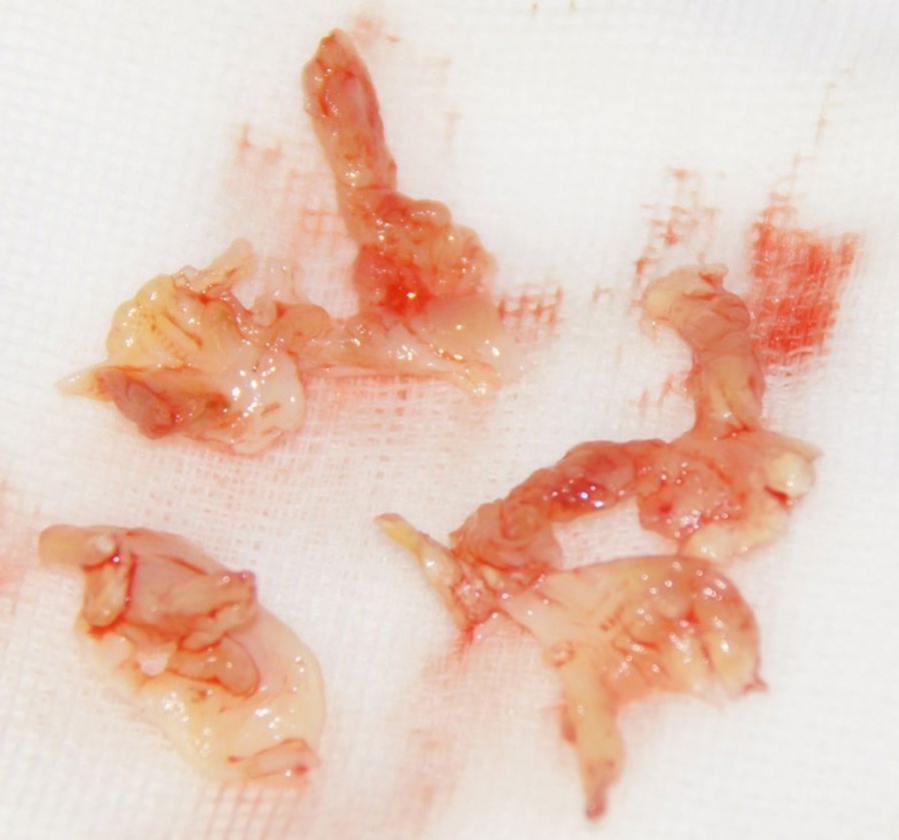
Three destroyed aortic valve leaflets with large vegetation

Considering the size of the emboli, emergent cardiac operation was performed. At surgery, a 3 × 6-mm aortic vegetation was found to have occluded the left main coronary trunk (Fig. 2), three aortic valve leaflets were destroyed (Fig. 3), and the mitral valve had multiple perforations at the anterior leaflet. Valve replacement with bioprosthetic valves (Carpentier-Edwards INSPIRIS 19 mm for the aortic valve and Medtronic Mosaic 25 mm for the mitral valve) and coronary artery bypass to the left antero-descending branch were performed. Regarding her cardiac function, she still required extracorporeal membrane oxygenation after the operation. She ultimately passed away 19 days after surgery due to multiple organ failure under extracorporeal membrane oxygenation.

## Discussion

ACS is a rare complication of IE. Causes of ACS are roughly divided into three reasons. The most common cause is septic embolization of an affected coronary artery. The second-most common cause is external compression of the coronary artery by an abscess. The least common cause is mechanical obstruction of the coronary artery by a vegetation.

Roux et al. reported only 1 case of ACS caused by left main coronary ostium obstruction by a vegetation among 1210 patients with IE. Only eight total cases of ACS in IE induced by a mechanical obstruction of the coronary artery by a vegetation have been reported according to Pub Med Central (Table 1). All nine mechanical obstruction cases, including the present case, were aortic valve endocarditis. Six of the nine cases were left coronary artery occlusion with severe symptoms, including three sudden deaths. Although there have only been three cases of right coronary artery occlusion reported, two of the cases survived after surgery.

**Table 1:** Eight cases of ACS in IE induced by a mechanical obstruction

Case type	Occluded coronary artery		Alive or death	IE affected valve	Conducted surgery	Symptoms	Age/Sex	Author [Ref.]
Surgical case	Right		Alive	Aortic, Mitral	Aortic/mitral valve replacement	STEMI	47/F	Casazza [1]
			Alive	Aortic	Aortic valve debriedment + PCI	STEMI → Vf arrest	53/F	Bolton [2]
	Left	Left coronary ostium partial occlusion	Alive	Aortic	Aortic valve replacement, mitral valve annuloplasty	Af/acute plmonary edema	43/F	Kim [3]
		LMT-proximal LAD, LCx ostium	Alive	Aortic	Aortic valve replacement + coronary bypass grafting	NSTEMI with plmonary edema	66/M	Pavani [4]
		LMT	Death	Aortic, mitral	Aortic/mitral valve replacement + coronary bypass grafting	Asystole	68/F	Our case
Autopsy case	Right		Death	Aortic			49/M	Ro [5]
	Left	LMT	Death	Aortic		Abdominal pain	44/M	Dowling [6]
		LMT	Death	Aortic			21/F	Fernando [7]
–		–	–	Bioprosthetic aortic valve			–	Roux [8]

*LMT* left main coronary trunk, *STEMI* ST-elevation myocardial infarction, *F* female, *M* male

As the initial manifestation of occlusion of the left main coronary artery is commonly sudden death, the diagnosis is usually made by a postmortem pathological examination. ACS caused by mechanical obstruction of the coronary artery with a vegetation is extremely rare but associated with high mortality and morbidity rates. The optimal management strategy of this condition remains controversial because of its scarcity. In cases of coronary obstruction with a big vegetation, catheter intervention is difficult due to its size. Surgery may thus be the only choice for revascularization. Mechanical removal of vegetations is performed with direct incision of the occluded coronary artery. However, evidence of successful surgical intervention in the context of coronary embolization is scarce, with only four cases demonstrating surgical success. Two cases successfully underwent aortic valve replacement without coronary bypass grafting. One case of right coronary artery occlusion underwent aortic valve debridement without bypass grafting but needed additional percutaneous coronary intervention after surgery. One case of left coronary artery occlusion underwent aortic valve replacement with double aorto-coronary bypass for the left antero-descending branch and the first obtuse marginal branch.

Coronary bypass grafting is theoretically not necessary after perfect coronary embolectomy; however, it may be required in cases with deterioration of the cardiac function. It must be kept in mind that mechanical obstruction of the coronary arteries by a vegetation can cause ACS.

Enterococcal species are the third most common cause of IE and are responsible for 10% of all cases of endocarditis. Enterococcal endocarditis was most frequently seen in elderly men, frequently involved the aortic valve, tended to produce heart failure rather than embolic events, and had relatively low short-term mortality. Though enterococcal endocarditis was associated with lower mortality among patients with left-sided native valve endocarditis, ACS caused by mechanical obstruction of the coronary artery with a vegetation associated with high mortality.

## Conclusion

ACS caused by mechanical obstruction of the coronary artery with a vegetation is extremely rare but associated with high mortality and morbidity rates. When circulatory collapse acutely occurs in patients with aortic valve IE, we should suspect acute coronary artery obstruction. Urgent coronary angiography is mandatory to rescue such patients while preparing for emergency surgery.

## Data Availability

All available data are presented in the main manuscript.

## Abbreviations

ACS: Acute coronary syndrome
IE: Endocarditis
MRI: Magnetic resonance imaging
